# Using UNICEF's Early Child Development Index 2030 to Identify Young Children With Significant Cognitive Delay

**DOI:** 10.1111/jir.13245

**Published:** 2025-04-24

**Authors:** Eric Emerson, Gwynnyth Llewellyn

**Affiliations:** ^1^ Centre for Disability Research, Faculty of Health & Medicine Lancaster University Lancaster UK; ^2^ Centre for Disability Research and Policy, Faculty of Medicine and Health University of Sydney Sydney New South Wales Australia; ^3^ Honorary Professorial Fellow, Melbourne School of Population and Global Health University of Melbourne Melbourne Australia

**Keywords:** cognitive behaviour, intellectual disability, methodology in research, (global) developmental delay

## Abstract

**Background:**

To help redress the global bias of intellectual disability research drawing on high‐income countries, previous studies have used data from UNICEF's Early Child Development Index (ECDI) to create an indicator of Significant Cognitive Delay (SCD) in young children. Recently, UNICEF have replaced the ECDI with an updated 20‐item version; the ECDI2030. Our aim was to investigate the utility of using ECDI2030 data to provide a more robust measure of SCD.

**Method:**

We conducted secondary analysis of ECDI2030 data on 92 506 2–4‐year‐old children from 23 nationally representative surveys undertaken primarily in the world's poorer countries.

**Results:**

The 11 learning items of the ECDI2030 showed good internal consistency overall and in each of the participating countries. Using age‐specific cut‐points for SCD generated from 20 013 children in nine countries with high Human Development Index (HDI) scores produced country‐level estimates of the prevalence of SCD that ranged from 1.1% to 34.1%. These prevalence estimates showed a strong relationship with both country HDI score and national wealth. Increased within country risk of SCD was independently associated with male gender, lower relative household wealth, lower level of maternal education and non‐enrolment in early childhood educational programmes. Comparison with SCD based on the older ECDI indicated that the two versions correlated very highly, although the newer version produced slightly higher prevalence estimates than the previous version.

**Conclusion:**

The ECDI2030 is being used in Round 7 of UNICEF's Multiple Indicator Cluster Surveys which are currently underway in 46 countries and in a growing number of USAID funded Demographic Health Surveys. Individual‐level data from surveys are freely available to researchers. As data from these surveys begin to be released, they will provide a highly cost‐efficient way to redress the current bias in intellectual and developmental disabilities research toward high‐income countries and to understand the of children at risk of intellectual disability or global developmental delay in the world's poorer countries.

## 1 Introduction

Intellectual disability research is almost exclusively focused on the situation of people living in high‐income countries (Emerson, Fujiura and Hatton 2007). One possible approach to reducing this inequity is for researchers to make greater use of data collected by UN Agencies and national governments in low‐ and middle‐income countries (LMICs) as they monitor progress toward the achievement of the UN's Sustainable Development Goals (SDG; https://sdgs.un.org/#goal_section).

Particularly relevant for investigating possible intellectual disability among children are data collected by UNICEF in their Multiple Indicator Cluster Survey programme (MICS) and, more recently, the USAID funded Demographic Health Surveys programme (DHS). Established in 1995, MICS aims to provide support to LMICs to generate robust country‐specific data on the wellbeing of children and mothers (Khan and Hancioglu 2019; UNICEF 2015). In 2009 UNICEF introduced the Early Child Development Index (ECDI) into MICS to identify the proportion of three and four‐year‐old children who were developmentally ‘on track’. Emerson, Llewellyn and colleagues used ECDI data in a series of studies to identify three and four‐year‐old children with significant cognitive delay (SCD), a possible proxy indicator for intellectual disability or global developmental delay (World Health Organization and UNICEF 2023). These studies have investigated such issues as: the prevalence of SCD in over 70 LMICs (Emerson and Llewellyn 2023); the association between variations in national prevalence estimates and country characteristics (Emerson and Llewellyn 2023); the potential impact of a range of preventative interventions on the global prevalence of SCD (Emerson, Savage and Llewellyn 2018); aspects of the health and healthcare received by young children with SCD (Emerson and Savage 2017; Emerson, Savage and Llewellyn 2020; Savage and Emerson 2016); and to assess the validity of an alternative indicator of functional limitations in learning introduced in MICS6 (Emerson and Llewellyn 2021).

In an attempt to provide better data to monitor progress toward the achievement of SDG Indicator 4.2.1 (the proportion of children under 5 years of age who are developmentally on track in health, learning and psychosocial well‐being), UNICEF have replaced the 10‐item ECDI with an updated 20‐item version; the ECDI2030 (Cappa et al. 2021; Halpin et al. 2024; United Nations Children's Fund 2023).

The aim of the present paper was to investigate the possibility of using ECDI2030 data to provide a robust measure of SCD by: (1) evaluating the internal consistency of the 11 learning domain items in the ECDI2030; (2) generating cut‐points for the identification of SCD from ECDI2030 data; (3) assessing the strength of association between SCD derived from ECDI2030 data with three well‐established correlates of intellectual disability (child sex, relative household wealth and level of maternal education), a potential intervention strategy for reducing the risk of SCD (enrolment in early childhood educational programmes; Black et al. 2017) and by country development status; and (4) to compare prevalence estimates obtained by using SCD derived from ECDI2030 data with the previous version of SCD derived from ECDI data.

## 2 Method

### 2.1 Data and Sample

We undertook secondary analysis of nationally representative data collected in Rounds 6 (2017–2023) of MICS and, from 2021 to 2023, by DHS (https://www.dhsprogram.com). Following approval by UNICEF, MICS data were downloaded from http://mics.unicef.org/. Following approval from the DHS programme, data were downloaded from https://www.dhsprogram.com/data/available‐datasets.cfm. At the end of the download period (31 March 2025), data from 23 nationally representative surveys were available that included the ECDI2030 (17 from MICS, 6 from DHS).

Data used in the present paper were extracted from the MICS household module and the MICS module applied to all children under five living in the household and the DHS module applied to women aged 15–49 years. All questionnaires were translated into the local languages of each country and then back translated as a validity check. Both surveys used cluster sampling methods to derive samples representative of the national population of mothers and young children. Specific details of the sampling procedure and ethical review procedure used in each survey and for each country are available in country‐specific reports freely available at either http://mics.unicef.org/or https://dhsprogram.com/. The median response rate for children under five in the surveys was 96.9% (range 81.3%–99.3%). The analytic sample was comprised of data from 92 506 2–4‐year‐old children for who valid ECDI2030 data were available in 23 nationally representative surveys.

### 2.2 ECDI2030

The ECDI2030 is based on milestones that children are expected to achieve before the age of five years (Cappa et al. 2021; Halpin et al. 2024; United Nations Children's Fund 2023). Development of the ECDI2030 included in‐depth cognitive testing of potential questions in Bulgaria, India, Jamaica, Mexico, Uganda and the USA (Cappa et al. 2021) and piloting in Mexico and Palestine (Halpin et al. 2024). It contains three domains: learning (11 items); psychosocial wellbeing (5 items); and health (4 items). ECDI2030 data were collected on children in the age range 24–59 months in 20 surveys and 36–59 months only in 2 surveys (Afghanistan and Comoros). Given that the primary aim of the ECDI2030 is to provide data for SDG Indicator 4.2.1, ECDI2030 guidance recommends against reporting domain scores for that purpose (United Nations Children's Fund 2023). However, given that our aim was to examine the utility of ECDI2030 data for identifying children with SCD, we used all 11 items from the learning domain to identify children with SCD. All items are based on key informant (primarily maternal) report with simple binary (yes/no) response options (see Table 1). Complete ECDI2030 data on the 11 learning items were missing for 3.0% of children in the age range to which it was applied.

**TABLE 1:** ECDI2030 Learning domain items.

**ECD5**. Can (***name***) say 10 or more words, like ‘mama’ or ‘ball’?
**ECD6**. Can (***name***) speak using sentences of three or more words that go together, for example, ‘I want water’ or ‘The house is big’?
**ECD7.** Can (***name***) speak using sentences of five or more words that go together, for example, ‘The house is very big’?
**ECD8**. Can (***name***) correctly use any of the words ‘I,’ ‘you,’ ‘she,’ or ‘he,’ for example, ‘I want water’ or ‘He eats rice’?
**ECD9**. If you show (***name***) an object (***he/she***) knows well, such as a cup or animal, can (***he/she***) consistently name it? By consistently we mean that (***he/she***) uses the same word to refer to the same object, even if the word used is not fully correct.
**ECD10**. Can (***name***) recognize at least five letters of the alphabet?
**ECD11**. Can (***name***) write (***his/her***) name?
**ECD12**. Can (***name***) recognize all numbers from 1 to 5?
**ECD13**. If you ask (***name***) to give you three objects, such as three stones or three beans, does (***he/she***) give you the correct amount?
**ECD14.** Can (***name***) count 10 objects, for example, 10 fingers or 10 blocks, without mistakes?
**ECD15**. Can (***name***) do an activity, such as colouring or playing with building blocks, without repeatedly asking for help or giving up too quickly?

### 2.3 Covariates

#### 2.3.1 Child Demographics

Information on child age (in months) and sex (male/female) was available for all children with valid ECDI2030 data.

#### 2.3.2 Household Wealth

Relative household wealth has been shown to be associated with variation in child development as measured by the ECDI (Gil et al. 2020). MICS and DHS data includes a within‐country wealth index for each household. To construct the wealth index, principal components analysis is performed by using information on the ownership of consumer goods, dwelling characteristics, water and sanitation and other characteristics that are related to the household's wealth, to generate weights for each item. Each household is assigned a wealth score based on the assets owned by that household weighted by factors scores. The wealth index is assumed to capture underlying long‐term wealth through information on the household assets (Howe et al. 2012; Rutstein 2008; Rutstein and Johnson 2004). Data were available for all children with valid ECDI2030 data.

#### 2.3.3 Maternal Education

Level of maternal education has been shown to be associated with variation in child development as measured by the ECDI (Gil et al. 2020). The highest level of education received by the child's mother was recorded using country‐specific categories. We recoded these data into a three‐category measure: (1) no or pre‐primary education; (2) primary education; (3) secondary or higher‐level education. Data were missing for 0.6% of children for who valid ECDI2030 data were available.

#### 2.3.4 Enrolment in Early Childhood Educational Programmes

Information was collected in the 17 MICS surveys for children aged 3 and 4 years on whether they had ever been enrolled in an early childhood educational programme. No information was available on the nature or quality of the programme. Data were missing for 0.1% of 3–4‐year‐old children for who valid ECDI2030 data were available.

#### 2.3.5 Country Characteristics

We sourced two measures of country characteristics that have been related to variations in child development.

Human Development Index (HDI): The HDI integrates three dimensions of human development: life expectancy at birth; mean years of schooling and expected years of schooling; and gross national income per capita (Anand and Sen 1994). HDI data for 2021 were taken from the 2021/22 Human Development Report (United Nations Development Programme 2022).

National wealth: Given the commonly reported association between child wellbeing and national wealth in LMICS (World Health Organization 2008), we used World Bank 2021 country classification as high‐income, upper‐middle income, lower‐middle income and low‐income (World Bank 2021c). These classifications are based on per capita Gross National Income (pcGNI; expressed as current US$ rates) using the World Bank's Atlas Method. We downloaded 2021 Atlas Method pcGNI from the World Bank website (World Bank 2021a, 2021b). HDI and pcGNI data were available for all countries.

### 2.4 Approach to Analysis

In the first stage of analysis, we investigated the internal consistency of the 11 items in the learning domain using McDonald's omega (McNeish 2018). Internal consistency was assessed separately for each country and, using pooled data across countries, separately for boys and girls, for each year of age and for each quartile of relative household income. We used the commonly used criteria of omega  >  0.7 to denote acceptable levels of internal consistency (McNeish 2018). These analyses were undertaken in SPSS 28 using country‐specific child‐level weights provided by UNICEF to take account of biases in sampling frames and household and individual‐level non‐response.

In the second stage of analysis, we followed the procedure used in ECDI2030 scoring (Halpin et al. 2024) to generate cut‐points in the summed scale for identifying children as not being developmentally ‘on track’ in relation to learning for five age groups (24–29, 30–35, 36–41, 42–47, 48–59 months). Previous studies suggest that approximately 2%–3% of children would be expected to have an IQ in the intellectual disability range in countries with ‘very high’ levels of human development (Maulik et al. 2011; McBride et al. 2020; Zablotsky et al. 2023). Given we only had access to data in two countries with very high levels of human development, we used the summed scale of the 11 learning domain items to generate cut‐points that would identify approximately 3%–5% of children in each age group as having SCD in the nine countries with ‘high’ or ‘very high’ levels of human development. The choice of a 3%–5% target constituted an adjustment to reflect the higher incidence of SCD in countries with high (when compared to very high) levels of human development (Emerson and Llewellyn 2023).

In the third stage of analysis, we applied these cut points to all available country surveys and assessed the extent to which prevalence varied by three factors known to be related to the prevalence of intellectual disability among children (child sex, relative household wealth and level of maternal education) and by a potential intervention (enrolment in an early childhood educational programme). These analyses involved mixed effects multilevel Poisson regression to estimate adjusted prevalence rate ratios (APRR) of the relationship between each predictor and SCD aggregated across countries with random effects specified to allow the intercept and slope for the association between the predictor and SCD to vary across countries. Mixed effects modelling was undertaken using the mepoisson command in Stata 16.1 with integration using mean–variance adaptive Gauss–Hermite quadrature to generate APRRs. We also investigated the correlation (Spearman's rho) between country‐level prevalence of SCD and country‐level HDI and pcGNI and country‐level enrolment rates into early childhood educational programmes.

In the final stage of analyses, we compared the prevalence of SCD derived from ECDI2030 data with that derived from ECDI data for the 10 countries in which we had access to previous estimates of SCD (Emerson and Llewellyn 2023). Stage three and four analyses were undertaken using Stata 16.1 using the UNICEF's country‐specific child‐level weights to take account of biases in sampling frames and household and individual‐level non‐response. Given the small amount of missing data, complete case analysis was undertaken.

## 3 Results

### 3.1 Internal Consistency

Country‐level results are presented in Table 2 along with information on survey characteristics (e.g., sample size) and national characteristics (e.g., national wealth). Internal consistency ranged from 0.725 (in Afghanistan) to 0.862 (in Nauru) with a mean of 0.812 (95% CI 0.797–0.827).

**TABLE 2:** Internal consistency of 11 learning domain items and survey characteristics by country.

Country	Year of survey	HDI 2021	Survey programme	pcGNI 2021	Sample size	Response rate	Internal consistency (omega)	Prevalence of SCD derived from ECDI2030 data	Prevalence of SCD previously derived from ECDI data
*Very High Human Development*									
Trinidad and Tobago	2022	0.810	MICS	$14 850	1141	87.7%	0.831	2.2% (1.2–4.2)	0.6% (0.2–1.7)
Thailand	2022	0.800	MICS	$7090	6962	93.5%	0.841	4.2% (3.2–5.6)	0.4% (0.2–0.6)
*High Human Development*									
Azerbaijan	2023	0.738	MICS	$4910	1596	92.5%	0.736	4.5% (3.3–6.0)	N/A
Jordan	2023	0.736	DHS	$4210	3304	96.9%	0.837	4.9% (3.7–6.5)	N/A
Tunisia	2023	0.731	MICS	$3560	1245	95.0%	0.846	4.0% (2.9–5.4)	4.1% (3.2–5.2)
Fiji	2021	0.730	MICS	$4500	1074	97.5%	0.832	5.1% (3.8–6.8)	N/A
Uzbekistan	2021/22	0.727	MICS	$1980	1159	99.3%	0.829	4.0% (2.9–5.5)	N/A
Jamaica	2022	0.709	MICS	$5190	830	92.8%	0.832	1.0% (0.5–1.8)	0.5% (0.2–1.4)
Viet Nam	2020/21	0.703	MICS	$3590	2535	97.2%	0.804	4.3% (3.3–5.5)	3.0% (2.1–4.3)
*Medium Human Development*									
Philippines	2022	0.699	DHS	$3550	4561	98.0%	0.812	5.8% (4.9–6.9)	N/A
Kyrgyzstan	2023	0.696	MICS	$1280	1857	97.6%	0.777	7.9% (6.5–9.6)	5.5% (4.4–6.8)
Nauru	2023	0.693	MICS	$18 010	201	81.3%	0.862	8.8% (5.4–14.1)	N/A
Lao PDR	2023	0.607	MICS	$2500	5155	98.7%	0.806	12.0% (10.9–13.3)	2.3% (1.9–2.8)
Vanuatu	2023	0.607	MICS	$3350	1256	93.8%	0.839	7.7% (5.9–10.0)	N/A
Eswatini	2021/22	0.597	MICS	$3740	1322	93.1%	0.763	10.6% (8.7–12.9)	3.8% (2.7–5.1)
Kenya	2022	0.575	DHS	$2080	5423	94.8%	0.840	5.5% (4.8–6.3)	N/A
Comoros	2022	0.558	MICS	$1580	1767	95.3%	0.839	27.9% (24.6–31.4)	N/A
Cote d'Ivoire	2021	0.550	DHS	$2470	5480	98.1%	0.770	27.0% (25.1–28.9)	10.7% (7.4–15.1)
*Low Human Development*									
Tanzania	2022	0.549	DHS	$1120	5529	96.9%	0.808	25.5% (23.6–27.4)	N/A
Nigeria	2020/21	0.535	MICS	$2120	16 459	97.9%	0.820	17.3% (16.3–18.3)	15.8% (14.6–18.3)
Benin	2021/22	0.525	MICS	$1330	7796	98.4%	0.810	28.2% (26.4–30.0)	19.6% (17.5–21.8)
Afghanistan	2022/23	0.478	MICS	$390	13 500	98.8%	0.725	19.1% (18.0–20.2)	N/A
Mozambique	2022/23	0.473	DHS	$490	2277	94.3%	0.821	34.1% (30.9–37.4)	N/A

*Note:* Sample size based on number of children with complete ECDI2030 data on the 11 learning domain items.

Abbreviations: HDI: Human Development Index, pcGNI: per capital gross national income.

Internal consistency showed: modest increases with age (age 2: 0.791, age 3: 0.798, age 4: 0.825), relative household wealth (income Q1: 0.818, income Q2: 0.823, income Q3: 0.822, income Q4: 0.827, income Q5: 0.833) and level of maternal education (none/primary: 0.786, secondary: 0.820, higher: 0.835). There was no variation by child sex (boys: 0.829, girls: 0.831). Internal consistency was not significantly related to country HDI score (*r* = 0.32, 95%CI –0.12 to 0.65) or national wealth (*r* = 0.37, 95%CI –0.06 to 0.69), but did exhibit a moderate effect size with both.

### 3.2 Cut‐Points for the Prevalence of SCD

Pooled cumulative pass rates for the 11 ECDI2030 items among children in very high or high human development countries for the age bands 24–29, 30–35, 36–41, 42–47 and 48–59 months are presented in Table 3. As can be seen, for each age band there was only one pass rate that fell within the 3%–5% target range: zero for children aged 24–29 and 30–35 months; two for children aged 36–41 and 42–47 months; and four for children aged 48–59 months. Applying these cut points to each country produced prevalence estimates for SCD ranging from 1.0% (Jamaica) to 34.1% (Mozambique) (Table 2). Endorsement patterns for the 11 ECDI2030 learning items by country and human development group are presented in a Table S1. Simple pooled estimates of SCD prevalence were 4.0% (95%CI 3.0–5.2) for very high human development countries, 4.3% (95%CI 3.8–4.8) for high human development countries, 13.2% (95%CI 12.6–13.9) for medium human development countries and 21.5% (95%CI 20.8–22.2) for low human development countries.

**TABLE 3:** Pooled cumulative pass rates for the 11 ECDI2030 items among children in very high or high human development countries.

	Number of items passed					
Age band (months)	0	1	2	3	4	5
24–29	5.3%	>10%	>10%	>10%	>10%	>10%
30–35	4.2%	8.8%	>10%	>10%	>10%	>10%
36–41	0.9%	2.0%	4.7%	8.1%	>10%	>10%
42–47	0.6%	1.6%	3.4%	6.2%	>10%	>10%
48–59	0.4%	0.9%	1.4%	2.2%	3.6%	7.3%

*Note:* Shaded cells denote cumulative pass rates in the target 3%–5% range.

**FIGURE 1:**
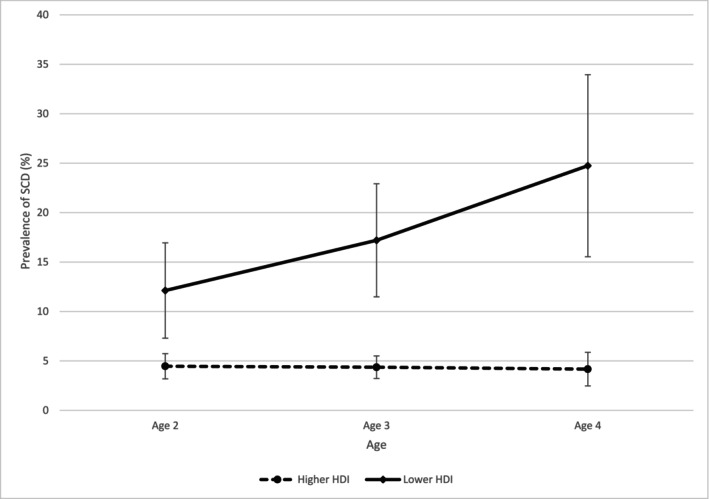
Percentage prevalence of SCD across age (with 95% confidence intervals) for the 11 highest HDI countries and the 10 lowest HDI countries.

Country‐level prevalence rates for SCD were highly correlated across ages (age 2–3 *r* = +0.93, *p* < 0.001; age 3–4 *r* = +0.96, *p* < 0.001). However, prevalence rates for SCD significantly increased with increasing age (age 2 *x̄*=8.1%, age 3 *x̄*=10.1%, *t* = −2.31, *p* < 0.05; age 4 *x̄*=13.8%, age 3 to 4 *t* = −2.51, *p* < 0.05). Visual inspection of these age differences suggested that they were primarily driven by increasing prevalence of SCD with age in lower HDI countries. To investigate further we examined prevalence rates over age separately for the 11 highest HDI countries and the 10 lowest HDI countries (Figure 1). For higher HDI countries the mean prevalence of SCD remained reasonably constant across ages (age 2 *x̄*=4.5%, age 3 *x̄*=4.4%, age 4 *x̄*=4.2%). In contrast, for lower HDI countries the mean prevalence of SCD systematically increased across ages (age 2 *x̄*=12.1%, age 3 *x̄*=17.2%, age 4 *x̄*=24.7%) resulting in a marked increase in inequality between country groups with increasing child age.

### 3.3 Variation in the Prevalence of SCD

National prevalence rates for SCD showed: (1) a strong relationship with HDI score (rho = −0.78) (95% CI −0.90 to −0.53, *p* < 0.001) and country wealth (rho = −0.67) (95% CI −0.85 to −0.35, *p* < 0.001) and (2) a non‐significant but modest inverse relationship with country‐level enrolment rates in early childhood educational programmes at age 3 (rho = −0.39) (95% CI −0.74 to +0.13) and at age 4 (rho = −0.44) (95% CI −0.77 to +0.06). Increased within country risk of SCD was independently associated with male gender, lower relative household income, lower level of maternal education and never having been enrolled in an early childhood education programme (Table 4). To illustrate the potential impact of enrolment in an early childhood education programme we have provided in Table 5 simple pooled estimates of SCD prevalence among 3–4 year old children who had been/never been enrolled in an early childhood education programme for each human development group of countries. Overall, children in low human development countries are 7.0 times more likely than their peers in very high human development countries to be identified as having SCD. Among children who had been enrolled in an early childhood education programme, children in low human development countries are 5.3 times more likely than their peers in very high human development countries to be identified as having SCD.

**TABLE 4:** Association between child sex, household wealth, level of maternal education and enrolment in early childhood educational programmes with risk of SCD for children aged 3–4 years.

Bivariate associations	Bivariate associations PRR (with 95% CI)	Multivariate associations PRR (with 95% CI)
Sex		
Male	1.11 (1.07–1.15)***	1.08 (1.05–1.12)***
Female (reference group)	1.00	1.00
Household wealth		
Poorest quintile	2.88 (2.11–3.94)***	2.05 (1.77–2.37)***
Quintile 2	2.36 (1.77–3.13)***	1.69 (1.37–2.10)***
Quintile 3	1.99 (1.62–2.44)***	1.57 (1.29–1.91)***
Quintile 4	1.51 (1.27–1.79)***	1.34 (1.11–1.63)**
Richest quintile (reference group)	1.00	1.00
Highest level of maternal education		
None/pre‐primary	2.71 (2.03–3.62)***	1.36 (1.22–1.52)***
Primary	1.66 (1.44–1.92)***	1.16 (1.01–1.33)*
Secondary or higher (reference group)	1.00	1.00
Enrolment in early childhood educational programmes		
Not enrolled	2.24 (1.85–2.71)***	1.75 (1.39–2.22)***
Enrolled (reference group)	1.00	1.00

*Note:* Bivariate associations estimated by mixed effects multilevel modelling. Multivariate associations estimated by fixed effects multilevel modelling.

* *p* < 0.05.

** *p* < 0.01.

*** *p* < 0.001.

**TABLE 5:** Simple pooled estimates of the prevalence of SCD were among 3–4‐year‐old children who had been/never been enrolled in an early childhood education programme for each human development group of countries.

	Very high human development	High human development	Medium human development	Low human development
Enrolled	2.3% (1.7–3.2)	2.5% (1.9–3.3)	7.6% (6.5–8.9)	12.2% (11.0–13.5)
Not enrolled	6.3% (4.0–9.9)	5.8% (4.7–7.0)	17.7% (16.3–19.1)	23.7% (22.8–254.6)
Total	3.3% (2.5–4.1)	4.2% (3.6–4.8)	15.7% (14.9–16.6)	23.2% (22.4–24.0)

### 3.4 Comparison With Previous Version of SCD

For 11 countries, SCD prevalence estimates derived from the ECDI2030 were compared to previous estimates of SCD derived from the ECDI (Table 2). The correlation between the two SCD measures was very strong *r* = 0.87 (95%CI 0.56–0.97, *p* < 0.01). However, the measure of SCD derived from the ECDI2030 gave higher prevalence estimates than the previous version (10.8% vs. 6.0%; *t* = 3.12, *p* < 0.01).

## 4 Discussion

Analyses of the cognitive development of 92 506 2–4‐year‐old children across 23 nationally representative surveys indicated that: (1) the 11 ‘learning’ items showed good internal consistency overall, in each of the participating countries and when analyses were stratified by child and family characteristics; (2) using cut‐points for SCD generated from the 20 013 2–4‐year‐old children in nine countries with high/very high HDI produced country‐level estimates of the prevalence of SCD that ranged from 1.1% to 34.1%; (3) these country‐level estimates of the prevalence of SCD showed a strong relationship with both HDI score and country wealth; (4) increased within country risk of SCD was independently associated with male gender, lower relative household wealth, lower level of maternal education and never having been enrolled in an early childhood education programme; and (5) comparison with a previous measure of SCD derived from the ECDI indicated that the two versions correlated very highly (*r* = 0.90), although the newer version produced higher prevalence estimates than the previous version (11.0% vs. 6.5%). The higher prevalence rates found in this study may be more accurate, since the previous measure of SCD produced lower than expected rates in upper‐middle income countries for example, less than 1% in 21/45 surveys in (Upper‐Middle Income countries) (Emerson and Llewellyn 2023).

The results suggest that very substantial inequalities in the prevalence of SCD are related to both country characteristics (level of human development and national wealth), living conditions (especially relative household wealth and level of maternal education) and enrolment in an early childhood education programme. These observations are consistent with those reported using an earlier version of SCD (Emerson and Llewellyn 2023) and research indicating that global estimates of loss of developmental potential in young children is primarily found in lower‐middle and low‐income countries and is associated with such factors as increased exposure to malnutrition, reduced access to early years education and increased risk of exposure to low levels of in‐home stimulation (Black et al. 2017; Emerson, Savage and Llewellyn 2018).

The primary limitation of our study lies in the unknown validity of our measure of SCD as a potential proxy indicator for risk of intellectual disability or global developmental delay. Circumstantial evidence of the potential validity of the measure is provided by: (1) the strength and direction of association between SCD and three well‐established correlates of intellectual disability (parental level of education, household wealth, male gender); and (2) increased country‐level prevalence rates were significantly associated with lower national wealth and lower HDI score (Durkin et al. 2007; Gil et al. 2020; Maulik et al. 2011; McCoy et al. 2016). Future validation of the measure of SCD should focus on direct comparisons between identification of SCD and intellectual disability or global developmental delay in a diverse range of countries and the investigation of longitudinal trajectories of SCD and the association between SCD and cognitive functioning and educational attainment in later childhood.

As of 5 April 2025, Round 7 of UNICEF's MICS is currently underway in 46 countries with eight surveys at the stage of data processing/analysis and three at the stage of data collection. When data from these surveys are released, they will provide a highly cost‐efficient opportunity for researchers working in the field of intellectual or developmental disabilities to examine the situation of children at risk of intellectual disability or global developmental delay in the world's LMICs. The free availability of the ECDI2030 also opens the possibility of its use in intervention studies that aim to reduce the loss of developmental potential of young children in the world's poorer countries.

## Conflicts of Interest

The authors declare no conflicts of interest.

## Supporting information


**Table S1.** Supporting information.

## Data Availability

The data that support the findings of this study are available from UNICEF. Restrictions apply to the availability of these data, which were used under license for this study. Data are available from https://mics.unicef.org/ with the permission of UNICEF.

## References

[jir13245-bib-0001] Anand, S. , and A. K. Sen . 1994. Human Development Index: Methodology and Measurement. United Nations Development Programme.

[jir13245-bib-0002] Black, M. M. , S. P. Walker , L. C. H. Fernald , et al. 2017. “Early Childhood Development Coming of Age: Science Through the Life Course.” Lancet 389, 77–90. 27717614 10.1016/S0140-6736(16)31389-7 PMC5884058

[jir13245-bib-0003] Cappa, C. , N. Petrowski , E. De Castro , et al. 2021. “Identifying and Minimizing Errors in the Measurement of Early Childhood Development: Lessons Learned From the Cognitive Testing of the ECDI2030.” International Journal of Environmental Research and Public Health 18, 12181. 10.3390/ijerph182212181. 34831937 PMC8618056

[jir13245-bib-0004] Durkin, M. S. , N. Schupf , Z. A. Stein , and M. W. Susser . 2007. “Childhood Cognitive Disability.” In Public Health and Preventive Medicine, edited by R. B. Wallace , 15th ed., 1173–1184. Appleton & Lange.

[jir13245-bib-0005] Emerson, E. , G. T. Fujiura , and C. Hatton . 2007. “International Perspectives.” In Handbook on Developmental Disabilities, edited by S. L. Odom , R. H. Horner , M. Snell , and J. Blacher . Guilford Press.

[jir13245-bib-0006] Emerson, E. , and G. Llewellyn . 2021. “Identifying Children at Risk of Intellectual Disability in UNICEF's Multiple indicator Cluster Surveys: Cross‐Sectional Survey.” Disability and Health Journal 14, 100986. 10.1016/j.dhjo.2020.100986. 32859553

[jir13245-bib-0007] Emerson, E. , and G. Llewellyn . 2023. “The Prevalence of Significant Cognitive Delay Among 3‐4‐Year‐Old Children Growing Up in Low‐ and Middle‐Income Countries: Results From 126 Nationally Representative Surveys Undertaken in 73 Countries.” Journal of Intellectual Disabilities Research 67, 1200–1215. 10.1016/j.dhjo.2022.101364. 36109168

[jir13245-bib-0008] Emerson, E. , and A. Savage . 2017. “Acute Respiratory Infection, Diarrhoea and Fever in Young Children at Risk of Intellectual Disability in 24 Low‐and Middle‐Income Countries.” Public Health 142, 85–93. 10.1016/j.puhe.2016.10.014. 28057204

[jir13245-bib-0009] Emerson, E. , A. Savage , and G. Llewellyn . 2018. “Significant Cognitive Delay Among 3‐ To 4‐Year Old Children in Low‐ and Middle‐Income Countries: Prevalence Estimates and Potential Impact of Preventative Interventions.” International Journal of Epidemiology 47, 1465–1474. 10.1093/ije/dyy161. 30085108

[jir13245-bib-0010] Emerson, E. , A. Savage , and G. Llewellyn . 2020. “Prevalence of Underweight, Wasting and Stunting Among Young Children With a Significant Cognitive Delay in 47 Low and Middle‐Income Countries.” Journal of Intellectual Disability Research 64, 93–102. 10.1111/jir.12698. 31845425

[jir13245-bib-0011] Gil, J. D. C. , F. Ewerling , L. Z. Ferreira , and A. J. D. Barros . 2020. “Early Childhood Suspected Developmental Delay in 63 Low‐ and Middle‐Income Countries: Large Within‐ and Between‐Country Inequalities Documented Using National Health Surveys.” Journal of Global Health 10, 010427. 10.7189/jogh.10.010427. PMC7295453 32566165

[jir13245-bib-0012] Halpin, P. , E. de Castro , N. Petrowski , and C. Cappa . 2024. “Monitoring Early Childhood Development at the Population Level: The ECDI2030.” Early Childhood Research Quarterly 67, 1–12. 10.1016/j.ecresq.2023.11.004.

[jir13245-bib-0013] Howe, L. D. , B. Galobardes , A. Matijasevich , et al. 2012. “Measuring Socio‐Economic Position for Epidemiological Studies in Low‐ and Middle‐Income Countries: A Methods of Measurement in Epidemiology Paper.” International Journal of Epidemiology 41, no. 3: 871–886. 10.1093/ije/dys037. 22438428 PMC3396323

[jir13245-bib-0014] Khan, S. , and A. Hancioglu . 2019. “Multiple Indicator Cluster Surveys: Delivering Robust Data on Children and Women Across the Globe.” Studies in Family Planning 50, no. 3: 279–286. 31486080 10.1111/sifp.12103 PMC6771654

[jir13245-bib-0015] Maulik, P. K. , M. N. Mascarenhas , C. D. Mathers , T. Dua , and S. Saxena . 2011. “Prevalence of Intellectual Disability: A Meta‐Analysis of Population‐Based Studies.” Research in Developmental Disabilities 32, 419–436. 10.1016/j.ridd.2010.12.018. 21236634

[jir13245-bib-0016] McBride, O. , P. Heslop , G. Glover , et al. 2020. “Prevalence Estimation of Intellectual Disability Using National Administrative and Household Survey Data: The Importance of Survey Question Specificity.” International Journal of Population Data Science 6, no. 1: 1342. 10.23889/ijpds.v6i1.1342. PMC8188522 34164584

[jir13245-bib-0017] McCoy, D. C. , E. D. Peet , M. Ezzati , et al. 2016. “Early Childhood Developmental Status in Low‐ and Middle‐Income Countries: National, Regional, and Global Prevalence Estimates Using Predictive Modelling.” PLoS Medicine 13, no. 6: e1002034. 10.1371/journal.pmed.1002034. 27270467 PMC4896459

[jir13245-bib-0018] McNeish, D. 2018. “Thanks Coefficient Alpha, We'll Take It From Here.” Psychological Methods 23, 412–433. 10.1037/met0000144. 28557467

[jir13245-bib-0019] Rutstein, S. O. 2008. “The DHS Wealth Index: Approaches for Rural and Urban Areas.” DHS Working Papers No. 60.

[jir13245-bib-0020] Rutstein, S. O. , and K. Johnson . 2004. “The DHS Wealth Index: DHS Comparative Reports No. 6.”

[jir13245-bib-0021] Savage, A. , and E. Emerson . 2016. “Overweight and Obesity Among Children at Risk of Intellectual Disability in 20 Low and Middle Income Countries.” Journal of Intellectual Disability Research 60, no. 11: 1128–1135. 10.1111/jir.12309. 27444252

[jir13245-bib-0022] UNICEF . 2015. “Monitoring the Situation of Children and Women for 20 Years: The Multiple Indicator Cluster Surveys (MICS) 1995–2015.”

[jir13245-bib-0023] United Nations Children's Fund . 2023. “The Early Childhood Development Index 2030: A New Measure of Early Childhood Development.”

[jir13245-bib-0024] United Nations Development Programme . 2022. “Human Development Report 2021/22. Uncertain Times, Unsettled Lives: Shaping Our Future in a Transforming World.”

[jir13245-bib-0025] World Bank . 2021a. “GNI Per Capita, Atlas Method (Current US$).” Accessed April–December, 2021a. http://data.worldbank.org/indicator/NY.GNP.PCAP.CD?view=chart.

[jir13245-bib-0026] World Bank . 2021b. “The World Bank Atlas Method ‐ Detailed Methodology.”Accessed April–December. https://datahelpdesk.worldbank.org/knowledgebase/articles/378832‐what‐is‐the‐world‐bank‐atlas‐method.

[jir13245-bib-0027] World Bank . 2021c. “World Bank Country and Lending Groups.” Accessed April–December. https://datahelpdesk.worldbank.org/knowledgebase/articles/906519‐world‐bank‐country‐and‐lending‐groups.

[jir13245-bib-0028] World Health Organization . 2008. Closing the Gap in a Generation: Health Equity Through Action on the Social Determinants of Health. Final Report of the Commission on the Social Determinants of Health. World Health Organization.

[jir13245-bib-0029] World Health Organization , and UNICEF . 2023. “Global Report on Children With Developmental Disabilities: From the Margins to the Mainstream.”

[jir13245-bib-0030] Zablotsky, B. , A. Ng , L. Black , and S. Blumberg 2023. “Diagnosed Developmental Disabilities in Children Aged 3–17 Years: United States, 2019–2021.” 37440277

