# Supplementary material for: Using UNICEF's Early Child Development Index 2030 to Identify Young Children With Significant Cognitive Delay
Source: J Intellect Disabil Res. 2025 Apr 24;69(7):621–9. doi: 10.1111/jir.13245 (PMC12198095; doi:10.1111/jir.13245)
Supplement: Supplementary file 1 — Table S1. Supporting information. [file JIR-69-621-s001.docx]

# Supplementary Table

| Endorsement patterns for the 11 ECDI learning items by country and human development group | | | | | | | | | | | |
| --- | --- | --- | --- | --- | --- | --- | --- | --- | --- | --- | --- |
|  | ECD5 | ECD6 | ECD7 | ECD8 | ECD9 | ECD10 | ECD11 | ECD12 | ECD13 | ECD14 | ECD15 |
| ***Very High Human Development*** | **90.6%** | **86.8%** | **70.8%** | **86.7%** | **89.3%** | **59.0%** | **12.1%** | **59.7%** | **61.3%** | **43.9%** | **70.3%** |
| Trinidad & Tobago | 94.8% | 88.5% | 75.9% | 85.8% | 94.5% | 83.3% | 27.0% | 78.9% | 79.1% | 64.7% | 85.30% |
| Thailand | 90.0% | 86.6% | 70.0% | 86.8% | 88.5% | 55.4% | 9.9% | 56.8% | 58.6% | 40.8% | 68.1% |
| ***High Human Development*** | **91.2%** | **89.4%** | **78.1%** | **85.9%** | **89.3%** | **37.9%** | **9.0%** | **54.6%** | **62.5%** | **47.1%** | **61.6%** |
| Azerbaijan | 93.7% | 87.6% | 72.3% | 89.6% | 88.8% | 15.8% | 2.3% | 33.2% | 53.7% | 35.0% | 54.6% |
| Jordan | 87.6% | 88.4% | 78.9% | 87.0% | 88.5% | 46.3% | 12.0% | 61.3% | 70.7% | 54.6% | 81.8% |
| Tunisia | 88.7% | 90.3% | 82.8% | 82.1% | 92.7% | 42.7% | 12.1% | 58.8% | 63.8% | 39.4% | 56.7% |
| Fiji | 88.2% | 86.6% | 74.7% | 82.6% | 88.3% | 52.9% | 12.6% | 55.3% | 57.0% | 37.0% | 63.4% |
| Uzbekistan | 90.9% | 89.6% | 69.4% | 83.3% | 89.4% | 27.3% | 1.9% | 72.6% | 61.1% | 49.1% | 45.1% |
| Jamaica | 95.8% | 93.2% | 82.8% | 87.6% | 95.8% | 73.8% | 26.7% | 70.5% | 68.5% | 58.9% | 80.2% |
| Viet Nam | 95.9% | 91.3% | 81.3% | 85.9% | 87.5% | 23.4% | 3.2% | 43.3% | 57.9% | 48.6% | 41.7% |
| ***Medium Human Development*** | **86.2%** | **83.4%** | **66.7%** | **78.9%** | **71.7%** | **28.8%** | **5.4%** | **34.4%** | **43.9%** | **24.5%** | **36.3%** |
| Philippines | 90.4% | 81.7% | 65.5% | 80.6% | 79.9% | 54.9% | 8.7% | 58.2% | 58.5% | 42.9% | 55.3% |
| Kyrgyzstan | 85.0% | 82.1% | 64.8% | 84.1% | 78.7% | 17.9% | 2.1% | 29.1% | 59.1% | 26.6% | 48.0% |
| Nauru | 86.0% | 80.3% | 72.0% | 82.9% | 78.8% | 56.0% | 10.9% | 58.0% | 64.3% | 40.4% | 57.0% |
| Lao PDR | 82.8% | 82.1% | 70.6% | 86.9% | 76.3% | 18.7% | 2.0% | 32.3% | 44.5% | 26.6% | 17.8% |
| Vanuatu | 90.2% | 85.6% | 76.2% | 86.8% | 72.4% | 34.6% | 9.7% | 39.2% | 53.0% | 33.3% | 53.0% |
| Eswatini | 91.1% | 88.0% | 71.8% | 72.8% | 66.3% | 17.3% | 1.6% | 18.6% | 20.7% | 6.8% | 21.6% |
| Kenya | 90.6% | 88.7% | 72.7% | 85.0% | 85.8% | 41.8% | 10.6% | 44.5% | 48.7% | 29.4% | 58.2% |
| Comoros | 80.4% | 81.3% | 68.5% | 64.9% | 59.3% | 35.7% | 6.0% | 42.8% | 42.7% | 22.8% | 18.5% |
| Cote d’Ivoire | 81.9% | 80.6% | 54.8% | 66.0% | 49.3% | 6.3% | 1.2% | 7.8% | 24.7% | 4.3% | 17.5% |
| ***Low Human Development*** | **80.2%** | **76.0%** | **59.4%** | **74.5%** | **65.9%** | **18.5%** | **2.7%** | **20.0%** | **35.4%** | **12.1%** | **23.0%** |
| Tanzania | 75.7% | 62.9% | 52.8% | 72.7% | 68.9% | 11.0% | 1.8% | 14.4% | 18.7% | 10.1% | 20.0% |
| Nigeria | 83.0% | 75.1% | 52.1% | 73.7% | 70.4% | 24.8% | 3.8% | 28.4% | 38.1% | 16.3% | 20.5% |
| Benin | 74.3% | 71.6% | 56.6% | 64.8% | 54.2% | 7.9% | 3.0% | 12.0% | 27.3% | 6.4% | 14.9% |
| Afghanistan | 86.0% | 86.6% | 74.0% | 83.7% | 69.2% | 22.1% | 1.6% | 18.6% | 45.0% | 11.6% | 31.6% |
| Mozambique | 56.9% | 67.7% | 52.4% | 64.2% | 46.1% | 7.1% | 1.8% | 8.4% | 27.8% | 9.9% | 23.8% |
| Notes: See Table 1 for the text of the ECDI2030 items | | | | | | | | | | | |
